# Some properties of branching processes with random control functions and affected by viral infectivity in random environments

**DOI:** 10.1186/s13662-023-03775-3

**Published:** 2023-05-15

**Authors:** Min Ren, Guanghui Zhang

**Affiliations:** College of Mathematics and Statistics, Suzhou University, Suzhou, 234000 China

**Keywords:** Branching processes, Random environments, Viral infectivity, Limit properties, Random control functions, Certainly extinct

## Abstract

In this paper, a model of branching processes with random control functions and affected by viral infectivity in independent and identically distributed random environments is established, and the Markov property of the model and a sufficient condition for the model to be certainly extinct under some conditions are discussed. Then, the limit properties of the model are studied. Under the normalization factor $\{S_{n}:n\in N\}$, the normalization processes $\{\hat{W}_{n}:n\in N\}$ are studied, and the sufficient conditions of $\{\hat{W}_{n}:n\in N\}$ a.s., $L^{1}$ and $L^{2}$ convergence are given; A sufficient condition and a necessary condition for convergence to a nondegenerate at zero random variable are obtained. Under the normalization factor $\{I_{n}:n\in N\}$, the normalization processes $\{\bar{W}_{n}:n\in N\}$ are studied, and the sufficient conditions of $\{\bar{W}_{n}:n\in N\}$ a.s., and $L^{1}$ convergence are obtained.

## Introduction

As an extension of the classical branching process, Sevast’yanov and Zubkov ([1]) established the branching process controlled by a real-valued function and studied the extinction and nonextinction probability of the model. Subsequently, Zubkov and Yanev ([2, 3]) generalized the model and established a controlled branching process with random control functions and discussed the conditions of extinction and nonextinction of the model. Yanev, Yanev, and Holzheimejr ([4–6]) studied some properties of the controlled branching processes in random environments, such as the extinction probability and extinction conditions. By using the properties of a conditional probability generating function, Bi and Li ([7]) obtained a sufficient condition for the inevitable extinction of a controlled branching process in random environments. Fang, Yang, and Li ([8]) studied the convergence rate of the limit of a normalized controlled branching process with random control function in random environments. Li, et al. ([9]) discussed the Markov property of a controlled branching process in random environments and the limit properties of the process after proper normalization, such as the conditions for convergence almost everywhere, convergence in $L^{1}$ and $L^{2}$. More research on controlled branching processes in random environments can be found in the literature ([10–14]). The reproduction process of species is affected by many factors such as natural environment and social environment, and highly infectious viruses such as the influenza virus, the SARS virus, and the novel coronavirus all have direct or indirect effects on the reproduction of species. Around 50 million people worldwide died of influenza in 1918, and according to the WHO, around 6.3 million people have died of the novel coronavirus as of June 30, 2022. Based on these issues, Ren et al. studied the Markov property of branching processes affected by viral infectivity in random environments, the limit properties of normalized processes, such as sufficient conditions for convergence almost everywhere and convergence in $L^{1}$ and the bisexual branching process affected by viral infectivity in random environments, and gave the Markov property of the model, the properties of the probability generating function, and the extinction condition of the processes ([15, 16]).

In this paper, we mainly study the Markov property, extinction probability, and some limit properties of the branching process with random control functions and affected by virus infectivity in random environments, and discuss the limit properties of the normalized processes $\{\hat{W}_{n},n\in N\}$ and $\{\bar{W}_{n},n\in N\}$, such as the conditions for convergence almost everywhere and convergence in $L^{1}$ and $L^{2}$.

The remainder of this paper is organized as follows. In Sect. 2, some notations, definitions, and conventions are introduced. Sections 3–6 are devoted to presenting the main results, including the Markov property, the extinction probability, and the limit properties.

## Preliminaries

In this section we present a convention, some notations, and basic definitions, which will be used in the remainder of the paper.

Let $(\Omega ,\mathfrak{F},P)$ be a probability space, $(\Theta ,\Sigma )$ a measurable space, $\vec{\xi}=\{\xi _{0},\xi _{1},\ldots \}$ an independent and identically distributed (i.i.d.) sequence of random variables mapping from $(\Omega ,\mathfrak{F},P)$ to $(\Theta ,\Sigma )$, and $N=\{0,1,2,\ldots \}$, $N^{+}=\{1,2,\ldots \}$. *T* is a shift operator such that $T(\vec{\xi})=\{\xi _{1},\xi _{2},\ldots \}$. $\{X_{nj},n\in N,j\in N^{+}\}$ is a cluster of random variables mapping from $(\Omega ,\mathfrak{F},P)$ to *N*. Let $\{P_{i}(\theta ):\theta \in \Theta , i\in N^{+}\}$, $\{Q(\theta ;k,i):\theta \in \Theta ,k,i\in N\}$ and $\{\alpha ^{x}(\theta )(1-\alpha (\theta ))^{1-x}, \theta \in \Theta ,x=0,1\}$ be probability distribution sequences. Let $\{\phi _{n}(k):n,k\in N\}$ be a cluster of i.i.d. random functions with respect to *n*, from *N* to *N*, with distribution $Q(\xi _{n};k,i)=P(\phi _{n}(k)=i|\vec{\xi})$, $i\in N$.

### Definition 2.1

If $\{Z_{n},n\in N\}$ satisfies

(i) $Z_{0}=N_{0}$, $Z_{n+1}=\sum_{j=1}^{\phi _{n}(Z_{n })}X_{nj}I_{nj}$, $n \in N,N_{0}$, $j\in N^{+}$;
(ii) $P(X_{nj}=r|\vec{\xi})=P_{r}(\xi _{n})$, $r, n\in N$, $j\in N^{+}$, $P(I_{nj}=x|\vec{\xi})=\alpha ^{x}(\xi _{n})(1-\alpha ( \xi _{n}))^{(1-x)}$, $x=0 \text{ or } 1$, $n\in N$, $j\in N^{+}$;
(iii) $P(X_{nj}=r_{nj},1\leq j\leq l,0\leq n\leq m|\vec{\xi})= \prod_{n=0}^{m}\prod_{j=1}^{l}P(X _{nj}=r_{nj}| \vec{\xi} )$, $r_{nj}\in N$, $1\leq j \leq l$, $0\leq n \leq m$, $m\in N$, $l\in N^{+}$;
(iv) for given $\overrightarrow{\xi}$, $\{X_{nj}:n\in N,j\in N^{+}\}$, $\{I_{nj}:n\in N,j\in N^{+}\}$ and $\{\phi _{n}(k):n,k\in N\}$ are of mutually conditional independence; furthermore, for given *n*, $\{(X_{nj},I_{nj}): j\in N^{+}\}$ is a sequence of i.i.d. two-dimensional random variables.

Then, $\{Z_{n}, n\in N\}$ is called a branching process with random control functions and affected by virus infectivity in random environments.

In the model under consideration, $X_{nj}$ represents the number of offspring produced by the *j*th particle in the *n*th generation. We set $I_{nj}=0$ when the *j*th particle in the *n*th generation dies of a viral infection, that is, it does not participate in the reproduction of the next generation; $I_{nj}=1$ means the *j*th particle in the *n*th generation does not have the virus or was cured of it, that is, it normally participates in the reproduction of the next generation, $\alpha (\xi _{n})$ represents the probability that the *n*th-generation particles will not be affected by the virus. $Z_{n+1}$ represents the total number of the $(n+1)$th-generation particles, $\phi _{n}(\cdot )$ represents the control function in the reproduction process of the *n*th-generation particles and $\phi _{n}(k)=i$ means that when the total number of the *n*th-generation particles is *k*, of which the number of particles participating in the reproduction of offspring is *i*.

We further introduce some convention and notations, which will be used in the following discussion.

In order to avoid the trivialities of the process, we assume throughout that

$(A_{1})$
For any $n\in N$, it holds that
  $$ 0< P_{0}(\xi _{n})+P_{1}(\xi _{n})< 1, \qquad 0< P\bigl(\phi _{n}(k)=k|\vec{\xi}\bigr)< 1,\quad \text{a.s.},k \in N^{+}. $$
$(A_{2})$
For any $n\in N$, it holds that
  $$ 0< \alpha (\xi _{n})< 1,\quad \text{a.s.} $$

Otherwise, if $\alpha (\xi _{n})=1$, a.s., for any $n\in N$, then the model under consideration will be the one in reference [9].

We give some notations by

$$\begin{aligned}& \mathfrak{F}_{n}(\vec{\xi})=\sigma (Z_{0},Z_{1}, \dots ,Z_{n}; \vec{\xi}),\qquad m(\xi _{n})=E(X_{n1}| \vec{\xi}),\qquad m_{2}(\xi _{n})=E\bigl(X_{n1}^{2}| \vec{\xi}\bigr),\\& \varepsilon (\xi _{n},Z_{n})=Z_{n}^{-1}E \bigl(\phi _{n}(Z_{n})| \vec{\xi}\bigr),\qquad \varepsilon (\xi _{n})=\sup_{Z_{n}\geq 0}\varepsilon ( \xi _{n},Z_{n}) , \qquad \varepsilon _{1}(\xi _{n})=\inf_{Z_{n}\geq 0} \varepsilon (\xi _{n},Z_{n}),\\& \varepsilon _{Z_{n}}(\xi _{n})=\varepsilon (\xi _{n})-\varepsilon ( \xi _{n},Z_{n}),\qquad \delta ^{2}(\xi _{n},k)=\operatorname{Var}\bigl(\phi _{n}(k)|\vec{\xi}\bigr) ,\\& \delta ^{2}(\xi _{n})=\sup_{k\geq 1}\delta ^{2}(\xi _{n},k),\quad n\in N ,k \in N^{+}.\\& A=\Biggl\{ (r_{l},d_{l}):\sum_{l=1}^{k}r_{l}d_{l}=j,r_{l} \in N,d_{l}=0 \text{ or } 1,l=1,2,\dots ,k,k\in N^{+}\Biggr\} . \end{aligned}$$

## Markov property

### Definition 3.1

If for any $x,n\in N$, it holds that

3.1 $$\begin{aligned}& P(X_{0}=x_{0}|\vec{\xi})=P(X_{0}=x_{0}| \xi _{0}), \end{aligned}$$

3.2 $$\begin{aligned}& P(X_{n+1}=x|X_{0},X_{1},\dots ,X_{n}, \vec{\xi})=P(\xi _{n};X_{n},x). \end{aligned}$$

Then, *X⃗* is defined as a Markov chain in random environment *ξ⃗*.

### Theorem 3.2

$\{Z_{n},n\geq 0\}$ *is a Markov chain in random environment* *ξ⃗* *with the one*-*step transition probabilities*

$$ P(\xi _{n};i,j)=\sum_{k=0}^{\infty}Q( \xi _{n};i,k )\cdot \sum_{(r_{l},d_{l})\in A}\prod _{l=1}^{k}p_{r_{l}}(\xi _{n}) \alpha ^{d_{l}}(\xi _{n}) \bigl(1-\alpha (\xi _{n})\bigr)^{(1-d_{l}) }. $$

### Proof

From the definition of $\{Z_{n},n\geq 0\}$, we have $P(Z_{0}=N_{0}|\vec{\xi})=P(Z_{0}=N_{0}|\xi _{0})$, namely equation (3.1) holds.

The following is to prove equation (3.2) is true. When *ξ⃗* is given, for any $n\in N$, $k\in N^{+}$, $\phi _{n}(k)$, $X_{nk}$ and $I_{nk}$ are mutually independent, hence we obtain, for any $i,j,i_{1},\ldots ,i_{n-1}\in N^{+}$,

$$\begin{aligned}& P(Z_{n+1}=j|Z_{0}=N_{0},Z_{1}=i_{1}, \dots ,Z_{n-1}=i_{n-1},Z_{n}=i, \vec{\xi}) \\& \quad = P\Biggl(\sum_{l=1}^{\phi _{n}(Z_{n})}X_{nl}I_{nl }=j \Big|Z_{0}=N_{0},Z_{1}=i_{1}, \dots ,Z_{n-1}=i_{n-1},Z_{n}=i,\vec{\xi}\Biggr) \\& \quad = \frac{P(\sum_{l=1}^{\phi _{n}(Z_{n})}X_{nl}I_{nl }=j ,Z_{0}=N_{0},Z_{1}=i_{1},\dots ,Z_{n-1}=i_{n-1},Z_{n}=i|\vec{\xi})}{P( Z_{0}=N_{0},Z_{1}=i_{1},\dots ,Z_{n-1}=i_{n-1},Z_{n}=i| \vec{\xi}) } \\& \quad = \sum_{k=0}^{\infty}P\Biggl(\sum _{l=1}^{\phi _{n}(i)}X_{nl}I_{nl}=j, \phi _{n}(i)=k\Big| \vec{\xi} \Biggr) \\& \quad = \sum_{k=0}^{\infty}Q(\xi _{n};i,k )\cdot \sum_{(r_{l},d_{l}) \in A }\prod _{l=1}^{k}p_{r_{l}}(\xi _{n})\alpha ^{d_{l}}(\xi _{n}) \bigl(1- \alpha (\xi _{n}) \bigr)^{(1-d_{l}) }. \end{aligned}$$

By Definition 3.1, it is immediately obvious that $\{Z_{n},n\geq 0\}$ is a Markov chain in random environment *ξ⃗* with one-step transition probabilities

$$ P(\xi _{n};i,j)=\sum_{k=0}^{\infty}Q( \xi _{n};i,k )\cdot \sum_{(r_{l},d_{l})\in A}\prod _{l=1}^{k}p_{r_{l}}(\xi _{n}) \alpha ^{d_{l}}(\xi _{n}) \bigl(1-\alpha (\xi _{n})\bigr)^{(1-d_{l}) }. $$

 □

### Lemma 3.3

*For any* $n\in N$, *it holds that*

(i) $E(Z_{n+1}|\mathfrak{F}_{n}(\vec{\xi}))=Z_{n}m(\xi _{n})\alpha ( \xi _{n})\varepsilon (\xi _{n},Z_{n})$ *a*.*s*.
  *In particular*, *it follows that*
  $$ N_{0}\prod_{i=0}^{n-1}m(\xi _{i})\alpha (\xi _{i})\varepsilon _{1}( \xi _{i})\leq E(Z_{n}|\vec{\xi})\leq N_{0}\prod _{i=0}^{n-1}m(\xi _{i}) \alpha (\xi _{i})\varepsilon (\xi _{i}). $$
(ii) $\operatorname{Var}(Z_{n+1}|\mathfrak{F}_{n}(\vec{\xi}))=Z_{n}\varepsilon (\xi _{n},Z_{n})\operatorname{Var}(X_{n1}I_{n1}| \vec{\xi})+m^{2}(\xi _{n})\alpha ^{2}(\xi _{n})\delta ^{2 }(\xi _{n},Z_{n})$.

### Proof

(i) Theorem 3.2 implies that

$$\begin{aligned} E\bigl(Z_{n+1}|\mathfrak{F}_{n}(\vec{\xi})\bigr) =&\sum _{j=0}^{\infty}jP(\xi _{n};Z_{n},j) \\ =&\sum_{j=0}^{\infty}j\cdot \sum _{k=0}^{\infty}Q(\xi _{n};Z_{n},k ) \cdot \sum_{(r_{l},d_{l})\in A}\prod_{l=1}^{k}p_{r_{l}}( \xi _{n})\alpha ^{d_{l}}(\xi _{n}) \bigl(1-\alpha ( \xi _{n})\bigr)^{(1-d_{l}) } \\ =&\sum_{k=0}^{\infty}Q(\xi _{n};Z_{n},k )\cdot \sum_{j=0}^{\infty}j \cdot \Biggl\{ \sum_{(r_{l},d_{l})\in A}\prod _{l=1}^{k}p_{r_{l}}( \xi _{n}) \alpha ^{d_{l}}(\xi _{n}) \bigl(1-\alpha (\xi _{n}) \bigr)^{(1-d_{l}) } \Biggr\} \\ =&\sum_{k=0}^{\infty}Q(\xi _{n};Z_{n},k )\cdot \sum_{j=0}^{\infty}j \cdot P\Biggl(\sum_{l=1}^{k} X_{nl}I_{nl}=j\Big|\vec{\xi}\Biggr) \\ =&\sum_{k=0}^{\infty}Q(\xi _{n};Z_{n},k )km(\xi _{n})\alpha (\xi _{n})=m( \xi _{n})\alpha (\xi _{n}) E\bigl(\phi _{n}(Z_{n})|\vec{\xi}\bigr) \\ =&Z_{n}m(\xi _{n})\alpha (\xi _{n})\varepsilon (\xi _{n},Z_{n}). \end{aligned}$$

Since

3.3 $$ E(Z_{n+1}|\vec{\xi})=E\bigl(E\bigl(Z_{n+1}| \mathfrak{F}_{n}( \vec{\xi})\bigr)|\vec{\xi}\bigr) =m(\xi _{n})\alpha (\xi _{n})\varepsilon (\xi _{n},Z_{n})E(Z_{n}| \vec{\xi}), $$

the recurrence relation of equation (3.3) gives

$$ E(Z_{n+1}|\vec{\xi})=N_{0}\prod _{i=0}^{n}m(\xi _{i})\alpha (\xi _{i}) \varepsilon (\xi _{i},Z_{i}). $$

By the definitions of $\varepsilon (\xi _{n})$ and $\varepsilon _{1}(\xi _{n})$, we deduce that

$$ N_{0}\prod_{i=0}^{n-1}m(\xi _{i})\alpha (\xi _{i})\varepsilon _{1}( \xi _{i} )\leq E(Z_{n}|\vec{\xi})\leq N_{0}\prod _{i=0}^{n-1}m(\xi _{i}) \alpha (\xi _{i})\varepsilon (\xi _{i}). $$

(ii) Using Theorem 3.2 gives

$$\begin{aligned} E\bigl(Z_{n+1}^{2}|\mathfrak{F}_{n}(\vec{\xi}) \bigr) =&\sum_{j=0}^{\infty}j^{2}P( \xi _{n};Z_{n},j) \\ =&\sum_{j=0}^{\infty}j^{2}\cdot \sum _{k=0}^{\infty}Q(\xi _{n};Z_{n},k )\sum_{(r_{l},d_{l})\in A}\prod_{l=1}^{k}p_{r_{l}}( \xi _{n}) \alpha ^{d_{l}}(\xi _{n}) \bigl(1-\alpha ( \xi _{n})\bigr)^{(1-d_{l}) } \\ =&\sum_{k=0}^{\infty}Q(\xi _{n};Z_{n},k )\sum_{j=0}^{\infty}j^{2 } \cdot P\Biggl(\sum_{l=1}^{k} X_{nl}I_{nl}=j\Big|\vec{\xi}\Biggr) \\ =&\sum_{k=0}^{\infty}Q(\xi _{n};Z_{n},k ) E\Biggl(\Biggl(\sum _{l=1}^{k}X_{nl}I_{nl} \Biggr)^{2}\Big|\vec{\xi}\Biggr) \\ =&\sum_{k=0}^{\infty}Q(\xi _{n};Z_{n},k )kE\bigl(X_{n1}^{2}I_{n1}^{2}| \vec{\xi}\bigr)+\sum_{k=0}^{\infty}Q(\xi _{n};Z_{n},k)k(k-1) \bigl(E(X_{n1}I_{n1}| \vec{\xi})\bigr)^{2} \\ =&\sum_{k=0}^{\infty}Q(\xi _{n};Z_{n},k )k\bigl\{ E\bigl(X_{n1}^{2}I_{n1}^{2}| \vec{\xi}\bigr)-\bigl(E(X_{n1}I_{n1 }|\vec{\xi}) \bigr)^{2}\bigr\} \\ &{}+\sum_{k=0}^{ \infty}Q( \xi _{n};Z_{n},k )k^{2}\bigl(m(\xi _{n})\alpha (\xi _{n} )\bigr)^{2} \\ =&Z_{n}\operatorname{Var}(X_{n1}I_{n1}|\vec{ \xi})\varepsilon (\xi _{n},Z_{n})+m^{2}( \xi _{n})\alpha ^{2}(\xi _{n}) \bigl(\delta ^{2}(\xi _{n},Z_{n})+Z_{n}^{2} \varepsilon ^{2}(\xi _{n},Z_{n})\bigr). \end{aligned}$$

Thus, it holds that

$$\begin{aligned} \operatorname{Var}\bigl(Z_{n+1}|\mathfrak{F}_{n}(\vec{\xi}) \bigr) =&E\bigl(Z_{n+1}^{2}|\mathfrak{F}_{n}( \vec{ \xi})\bigr)-(E\bigl(Z_{n+1}|\mathfrak{F}_{n}(\vec{\xi}) \bigr)^{2} \\ =&Z_{n}\varepsilon (\xi _{n},Z_{n}) \operatorname{Var}(X_{n1}I_{n1}|\vec{\xi})+m^{2}( \xi _{n})\alpha ^{2}(\xi _{n})\delta ^{2}(\xi _{n},Z_{n}). \end{aligned}$$

 □

## The extinction probability of $\{Z_{n},n\in N\}$

An important tool in the analysis of the branching process in random environments is the generating function. In order to discuss the extinction probability of the model, we first introduce the relevant conditional probability generating function of the model as follows

$$ \Pi _{\xi _{n}}(s)=E\bigl(s^{Z_{n}}|\vec{\xi},Z_{0}=N_{0} \bigr),\qquad f_{\xi _{n}}(s)=E\bigl(s^{X_{ni}}| \vec{\xi},Z_{0}=N_{0} \bigr),\quad n\in N,0\leq s\leq 1. $$

For any $n\in N$, $i\in N^{+}$, from the independence of $X_{ni}$ and $I_{ni}$, we obtain

$$ E\bigl(s^{X_{ni}I_{ni}}|\vec{\xi},Z_{0}=N_{0}\bigr)=1- \alpha (\xi _{n})+\alpha ( \xi _{n})f_{\xi _{n}}(s) $$

and we designate $B(w)=\{w:Z_{n}=0,n\in N^{+}\}$, $q(\vec{\xi})=P(B(w)|\vec{\xi},Z_{0}=N_{0})$ and $q=P(B(w)| Z_{0}=N_{0})$, then $q=E(q(\vec{\xi}))$.

If for some $n\in N$, $q=1$, then we say $\{Z_{n},n\in N\}$ is certainly extinct; otherwise, $\{Z_{n},n\in N\}$ is noncertainly extinct.

### Lemma 4.1

*If there exists a sequence of i*.*i*.*d*. *random variables* $\{\eta _{n},n\in N\}$ *such that for any* $n\in N$, $\sup_{k\geq 1}\frac{\phi _{n}(k)}{k}\leq \eta _{n}$ *a*.*s*., *then*

4.1 $$\begin{aligned} \Pi _{\xi _{n}}(s) \geq &\bigl\{ 1-\alpha (\xi _{0})+\alpha (\xi _{0})f_{ \xi _{0}}\bigl[\bigl(1-\alpha (\xi _{1})+\alpha (\xi _{1})f_{\xi _{1}}\bigl(\dots \bigl(1-\alpha (\xi _{n-1}) \\ &{}+\alpha (\xi _{n-1})f_{\xi _{n-1}}(s)\bigr)^{\eta _{n-1}}\cdots \bigr)\bigr)^{ \eta _{1}}\bigr]\bigr\} ^{\eta _{0}N_{0}}. \end{aligned}$$

### Proof

From the assumed condition, the properties of the generating functions of conditional probability and the fact that for any fixed *n*, $X_{nj}I_{nj}$ is i.i.d. with respect to *j*, it follows that

$$\begin{aligned} E\bigl(s^{Z_{1}}|\vec{\xi},Z_{0}=N_{0}\bigr) =& E \bigl(s^{\sum _{j=1}^{\phi _{0}(Z_{0})}X_{0j}I_{0j}}| \vec{\xi},Z_{0}=N_{0}\bigr) \\ =& E\bigl(\bigl(1-\alpha (\xi _{0})+\alpha (\xi _{0})f_{\xi _{0}}(s) \bigr)^{ \phi _{0}(N_{0})}|\vec{\xi}\bigr) \\ =&\bigl\{ 1-\alpha (\xi _{0})+\alpha (\xi _{0})f_{\xi _{0}}(s) \bigr\} ^{\phi _{0}(N_{0})} \\ \geq &\bigl\{ 1-\alpha (\xi _{0})+\alpha (\xi _{0})f_{\xi _{0}}(s) \bigr\} ^{ \eta _{0}N_{0}}, \end{aligned}$$

namely (4.1) holds for $n=1$. Supposing (4.1) holds for $n=k$, we deduce by induction, for $n=k+1$,

$$\begin{aligned} \Pi _{\xi _{k+1}}(s) =&E\bigl(E\bigl(s^{Z_{k+1}}|Z_{0}=N_{0},Z_{1}, \ldots ,Z_{k}, \vec{\xi}\bigr)|\vec{\xi},Z_{0}=N_{0} \bigr) \\ =&E\bigl(E\bigl(s^{\sum _{j=1}^{\phi _{k}(Z_{k})}X_{kj}I_{kj}}|Z_{0}=N_{0},Z_{1}, \ldots ,Z_{k},\vec{\xi}\bigr)|\vec{\xi},Z_{0}=N_{0} \bigr) \\ =&E\bigl(\bigl(1-\alpha (\xi _{k})+\alpha (\xi _{k})f_{\xi _{k}}(s) \bigr)^{\phi _{k}(Z_{k})}| \vec{\xi},Z_{0}=N_{0}\bigr) \\ \geq &E\bigl(\bigl(\bigl(1-\alpha (\xi _{k})+\alpha (\xi _{k})f_{\xi _{k}}(s)\bigr)^{ \eta _{k}}\bigr)^{Z_{k}}| \vec{\xi},Z_{0}=N_{0}\bigr) \\ \geq &\bigl\{ 1-\alpha (\xi _{0})+\alpha (\xi _{0})f_{\xi _{0}} \bigl[\bigl(1-\alpha ( \xi _{1})+\alpha (\xi _{1})f_{\xi _{1}} \bigl(\cdots \bigl(1-\alpha (\xi _{k}) \\ &{}+\alpha (\xi _{k})f_{\xi _{k}}(s)\bigr)^{\eta _{k}}\cdots \bigr)\bigr)^{\eta _{1}}\bigr] \bigr\} ^{\eta _{0}N_{0}}, \end{aligned}$$

namely (4.1) holds for $n=k+1$, which completes the proof of Lemma 4.1,

$$\begin{aligned} \mu _{n}(\vec{\xi},\vec{\eta}) =&\bigl\{ 1-\alpha (\xi _{0})+\alpha (\xi _{0})f_{ \xi _{0}}\bigl[\bigl(1-\alpha (\xi _{1})+\alpha (\xi _{1})f_{\xi _{1}}\bigl(\cdots \bigl(1- \alpha (\xi _{n-1}) \\ &{}+\alpha (\xi _{n-1})f_{\xi _{n-1}}(0)\bigr)^{\eta _{n-1}}\cdots \bigr)\bigr)^{ \eta _{1}}\bigr]\bigr\} ^{\eta _{0}N_{0}},\quad n\in N^{+}. \end{aligned}$$

By the properties of generating functions,

$$ 0\leq \mu _{n}(\vec{\xi},\vec{\eta})\leq \mu _{n+1}(\vec{ \xi}, \vec{\eta})\leq 1,\quad \text{a.s.} $$

and

$$ \mu _{n}(\vec{\xi},\vec{\eta})=\bigl[1-\alpha (\xi _{0})+ \alpha (\xi _{0})f_{ \xi _{0}}\bigl(\mu _{n-1}(T\vec{\xi},T \vec{\eta})\bigr)\bigr]^{\eta _{0}N_{0}}. $$

Thus, $\mu (\vec{\xi},\vec{\eta})=\lim_{n\rightarrow \infty}\mu _{n}( \vec{\xi},\vec{\eta})$ a.s., and

$$ \mu (\vec{\xi},\vec{\eta})=\bigl[1-\alpha (\xi _{0})+\alpha (\xi _{0})f_{ \xi _{0}}\bigl(\mu (T\vec{\xi},T\vec{\eta})\bigr) \bigr]^{\eta _{0}N_{0}}\quad\text{a.s.} $$

For $q(\vec{\xi})=\lim_{n\rightarrow \infty}\Pi _{\xi _{n}}(0)$, then by (4.1)

$$ q(\vec{\xi})\geq \mu (\vec{\xi},\vec{\eta}) \quad \text{a.s.} $$

 □

### Lemma 4.2

*Suppose for any* $n\in N$,

- *If there exists a sequence of i*.*i*.*d*. *random variables* $\{\eta _{n},n\in N\}$ *such that*
  $$ \sup_{k\geq 1}\frac{\phi _{n}(k)}{k}\leq \eta _{n}\quad \textit{a.s.}; $$
- $E((\log \eta _{0}N_{0}\alpha (\xi _{0})f'_{ \xi _{0}}(1))^{+})<\infty $ *and* $\frac{1-(1-\alpha (\xi _{0})+\alpha (\xi _{0})f_{\xi _{0}}(s))^{\eta _{0}N_{0}}}{1-s}$ *is strictly monotonically increasing with respect to* *s* *on* $(0,1]$.

*Then*, *on* $\{q(\vec{\xi})<1\} $, *it holds that*

(i) $E(| \log \frac{1-\mu (\vec{\xi},\vec{\eta})}{1-\mu (T\vec{\xi},T\vec{\eta})} | )<\infty $, $E(\log \frac{1-\mu (\vec{\xi},\vec{\eta})}{1-\mu (T\vec{\xi},T\vec{\eta})})=0$;
(ii) $E(| \log N_{0}\eta _{0}\alpha (\xi _{0})f'_{\xi _{0}}(1) | )<\infty $, $E(\log N_{0}\eta _{0}\alpha (\xi _{0})f'_{\xi _{0}}(1))>0$.

### Proof

To prove (i), by Lemma 4.1, we obtain

$$ q(\vec{\xi})\geq \mu (\vec{\xi},\vec{\eta}) \quad \text{a.s.}, $$

hence,

$$ \bigl\{ q(\vec{\xi})< 1\bigr\} \subset \bigl\{ \mu (\vec{\xi},\vec{\eta})< 1\bigr\} . $$

If

$$ P\bigl(q(\vec{\xi})< 1\bigr)>0, $$

then

$$ P\bigl(\mu (\vec{\xi},\vec{\eta})< 1\bigr)>0. $$

Denote

$$ h(\vec{\xi},\vec{\eta})=-\log \bigl(1-\mu (\vec{\xi},\vec{\eta})\bigr),\qquad f( \vec{ \xi},\vec{\eta})=-\log \frac{1-\mu (\vec{\xi},\vec{\eta})}{1-\mu (\vec{T\xi},\vec{T\eta})}, $$

then

$$ P\bigl(0< h(\vec{\xi},\vec{\eta})< \infty \bigr)>0. $$

Since

$$ -\log \bigl(1-\mu (\vec{\xi},\vec{\eta})\bigr)=-\log \frac{1-\mu (\vec{\xi},\vec{\eta})}{1-\mu (\vec{T\xi},\vec{T\eta})}- \log \bigl(1-\mu (T\vec{\xi},T\vec{\eta})\bigr), $$

then

$$ h(\vec{\xi},\vec{\eta})=f(\vec{\xi},\vec{\eta})+h(T\vec{\xi},T \vec{\eta}) $$

and iterating this gives

$$ h(\vec{\xi},\vec{\eta})=f(\vec{\xi},\vec{\eta})+f(T\vec{\xi},T \vec{\eta})+ \cdots +f\bigl(T^{n}\vec{\xi},T^{n}\vec{\eta}\bigr)+h \bigl(T^{n+1} \vec{\xi},T^{n+1}\vec{\eta}\bigr). $$

Hence, on {$q(\vec{\xi})<1$}, by the nonnegativity of $h(\vec{\xi},\vec{\eta})$, we arrive at

$$ \sum_{j=0}^{n}f\bigl(T^{j}\vec{ \xi},T^{j}\vec{\eta}\bigr)\leq h( \vec{\xi},\vec{\eta}), $$

i.e.,

4.2 $$ n^{-1}\Biggl\{ \sum_{j=0}^{n}f^{+} \bigl(T^{j}\vec{\xi},T^{j}\vec{\eta}\bigr)- \sum _{j=0}^{n}f^{-}\bigl(T^{j}\vec{ \xi}, T^{j}\vec{\eta}\bigr)\Biggr\} \leq n^{-1}h( \vec{\xi}, \vec{\eta}). $$

From the monotonicity of $\frac{1-[1-\alpha (\xi _{0})+\alpha (\xi _{0})f_{\xi _{0}}(s))]^{\eta _{0}N_{0}}}{1-s}$, it follows that

$$\begin{aligned} 0 \leq & E\bigl(f^{-}(\vec{\xi},\vec{\eta})\bigr)=E\bigl(-f(\vec{ \xi},\vec{\eta}),f( \vec{\xi},\vec{\eta})\leq 0\bigr) \\ =& E\biggl(\log \frac{1-[1-\alpha (\xi _{0})+\alpha (\xi _{0})f_{\xi _{0}}(\mu (T\vec{\xi},T\vec{\eta}))]^{\eta _{0}N_{0}}}{1-\mu (T\vec{\xi},T\vec{\eta})} ,\\ & \frac{1-[1-\alpha (\xi _{0})+\alpha (\xi _{0})f_{\xi _{0}}(\mu (T\vec{\xi},T\vec{\eta}))]^{\eta _{0}N_{0}}}{1-\mu (T\vec{\xi},T\vec{\eta})} \geq 1\biggr) \\ \leq &E\bigl(\log \eta _{0}N_{0}\alpha (\xi _{0})f'_{\xi _{0}}(1)\bigl[1- \alpha (\xi _{0})+\alpha (\xi _{0})f_{\xi _{0}}(1) \bigr]^{\eta _{0}N_{0}-1} , \eta _{0}N_{0}\alpha (\xi _{0})f'_{\xi _{0}}(1)\geq 1\bigr) \\ \leq &E\bigl(\log \eta _{0}N_{0}\alpha (\xi _{0})f'_{\xi _{0}}(1) , \eta _{0}N_{0} \alpha (\xi _{0})f'_{\xi _{0}}(1)\geq 1\bigr) \\ =&E\bigl(\bigl(\log \eta _{0}N_{0}\alpha (\xi _{0})f'_{\xi _{0}}(1)\bigr)^{+}\bigr)< \infty . \end{aligned}$$

On $\{q(\vec{\xi})<1\}$, it holds that $\lim_{n\rightarrow \infty}n^{-1}h(\vec{\xi},\vec{\eta})=0$. Since $(\vec{\xi},\vec{\eta})$ are i.i.d., according to (4.2), we arrive at

$$ 0\leq \limsup_{n\rightarrow \infty}n^{-1}\sum _{j=0}^{n}f^{+}\bigl(T^{j} \vec{ \xi},T^{j}\vec{\eta}\bigr)\leq E\bigl(\bigl(\log \eta _{0}N_{0}\alpha (\xi _{0})f'_{ \xi _{0}}(1) \bigr)^{+}\bigr)< \infty . $$

By the law of large numbers, we have $E(f^{+}(\vec{\xi},\vec{\eta}))<\infty $, so $E(|f(\vec{\xi},\vec{\eta})|)<\infty $. As

$$ E\bigl(f(\vec{\xi},\vec{\eta})\bigr)=\lim_{n\rightarrow \infty}n^{-1} \sum_{j=0}^{n}f\bigl(T^{j}\vec{ \xi},T^{j}\vec{\eta}\bigr) =\lim_{n\rightarrow \infty}n^{-1} \bigl\{ h(\vec{\xi},\vec{\eta})-h\bigl(T^{n+1} \vec{\xi},T^{n+1} \vec{\eta}\bigr)\bigr\} , $$

$\lim_{n\rightarrow \infty}n^{-1}h(\vec{\xi},\vec{\eta})=0$ and $(\vec{\xi},\vec{\eta})$ are i.i.d., then $\lim_{n\rightarrow \infty}n^{-1}h(T^{n+1}\vec{\xi},T^{n+1} \vec{\eta})=0$.

Thus, we have $E(f(\vec{\xi},\vec{\eta}))=0$, which completes the proof of (i).

Now, we turn to prove (ii). We only need to show that

$$ E\bigl(\bigl(\log \eta _{0}N_{0}\alpha (\xi _{0})f'_{\xi _{0}}(1)\bigr)^{-}\bigr)\leq E\bigl(\bigl( \log \eta _{0}N_{0}\alpha (\xi _{0})f'_{\xi _{0}}(1)\bigr)^{+}\bigr). $$

A direct calculation gives

$$\begin{aligned}& E\bigl(\bigl(\log \eta _{0}N_{0}\alpha (\xi _{0})f'_{\xi _{0}}(1)\bigr)^{-}\bigr) \\& \quad =E\bigl(-\log \eta _{0}N_{0}\alpha (\xi _{0})f'_{\xi _{0}}(1),\eta _{0}N_{0} \alpha (\xi _{0})f'_{\xi _{0}}(1)\leq 1\bigr) \\& \quad \leq E\biggl(-\log \frac{1-[1-\alpha (\xi _{0})+\alpha (\xi _{0})f_{\xi _{0}}(\mu (T\vec{\xi},T\vec{\eta}))]^{\eta _{0}N_{0}}}{1-\mu (T\vec{\xi},T\vec{\eta})}, \eta _{0}N_{0} \alpha (\xi _{0})f'_{\xi _{0}}(1)\leq 1\biggr) \\& \quad =E\biggl(-\log \frac{1-\mu (\vec{\xi},\vec{\eta})}{1-\mu (T\vec{\xi},T\vec{\eta})}, \eta _{0}N_{0} \alpha (\xi _{0})f'_{\xi _{0}}(1)\leq 1\biggr) \\& \quad \leq E\bigl(f(\vec{\xi},\vec{\eta}), f(\vec{\xi},\vec{\eta})\geq 0\bigr)=E \bigl(f^{+}( \vec{\xi},\vec{\eta})\bigr) \\& \quad \leq E\bigl(\bigl(\log \eta _{0}N_{0}\alpha (\xi _{0})f'_{\xi _{0}}(1)\bigr)^{+}\bigr)< \infty . \end{aligned}$$

If

$$ E\bigl(\bigl(\log \eta _{0}N_{0}\alpha (\xi _{0})f'_{\xi _{0}}(1)\bigr)^{+}\bigr)=E \bigl(\bigl( \log \eta _{0}N_{0}\alpha (\xi _{0})f'_{\xi _{0}}(1)\bigr)^{-}\bigr), $$

then

$$ E\bigl(\log \eta _{0}N_{0}\alpha (\xi _{0})f'_{\xi _{0}}(1) \bigr)=0 $$

and since $E(f(\vec{\xi},\vec{\eta}))=0$, then

$$ E\bigl(f(\vec{\xi},\vec{\eta})+\log \eta _{0}N_{0}\alpha ( \xi _{0})f'_{ \xi _{0}}(1)\bigr)=0. $$

From the assumed monotonicity it follows that

$$ P\bigl(f(\vec{\xi},\vec{\eta})+\log \eta _{0}N_{0}\alpha ( \xi _{0})f'_{ \xi _{0}}(1)\geq 0\bigr)=1, $$

and

$$ P\bigl(f(\vec{\xi},\vec{\eta})+\log \eta _{0}N_{0}\alpha ( \xi _{0})f'_{ \xi _{0}}(1)>0\bigr)>0, $$

unless $P(P_{1}(\xi _{n})=1)=1$, which contradicts with

$$ P\bigl(P_{0}(\xi _{n})+P_{1}(\xi _{n})< 1\bigr)=1. $$

Thus, it holds that

$$ E\bigl(\log \eta _{0}N_{0}\alpha (\xi _{0})f'_{\xi _{0}}(1) \bigr)>0. $$

 □

### Theorem 4.3

*Suppose for any* $n\in N$,

(i) *If there exists a sequence of i*.*i*.*d*. *random variables* $\{\eta _{n},n\in N\}$ *such that*
  $$ \sup_{k\geq 1}\frac{\phi _{n}(k)}{k}\leq \eta _{n}\quad \textit{a.s.}; $$
(ii) $E((\log \eta _{0}N_{0}\alpha (\xi _{0})f'_{\xi _{0}}(1))^{+})< \infty $ *and* $\frac{1-(1-\alpha (\xi _{0})+\alpha (\xi _{0})f_{\xi _{0}}(s))^{\eta _{0}N_{0}}}{1-s}$ *is strictly monotonically increasing with respect to* *s* *on* $(0,1]$.

*Then*, *when* $E((\log \eta _{0}N_{0}\alpha (\xi _{0})f'_{\xi _{0}}(1)))\leq 0$, *we have* $P(q(\vec{\xi})=1)=1$, *i*.*e*., $\{Z_{n},n\in N\}$ *is certainly extinct*.

### Proof

We proceed with the proof by contradiction. Suppose $P(q(\vec{\xi})=1)<1$ when $E((\log \eta _{0}N_{0}\alpha (\xi _{0})f'_{\xi _{0}}(1)))\leq 0$, then

$$ P\bigl(q(\vec{\xi})< 1\bigr)=1-P\bigl(q(\vec{\xi})=1\bigr)>0. $$

From Lemma 4.2 we obtain that the assumed conditions (i) and (ii) in this theorem hold, then on $\{q(\vec{\xi})<1\}$,

$$ E\bigl(\bigl(\log \eta _{0}N_{0}\alpha (\xi _{0})f'_{\xi _{0}}(1)\bigr)\bigr)>0, $$

which contradicts our assumption and completes the proof. □

Since the expression of the conditional expectation of the process cannot be calculated precisely, using the upper and lower bounds of the conditional expectation of the process given by Lemma 3.3, we define two random sequences $\{S_{n},n\in N\}$ and $\{I_{n},n\in N\}$, where

$$ S_{n}=N_{0}\prod_{k=0}^{n-1}m( \xi _{k})\alpha (\xi _{k})\varepsilon ( \xi _{k}),\qquad I_{n}=N_{0}\prod _{k=0}^{n-1}m(\xi _{k})\alpha (\xi _{k}) \varepsilon _{1}(\xi _{k}),\quad n\in N^{+}, $$

and obviously $S_{0}=I_{0}=N_{0}$. Regarding $S_{n}$ and $I_{n}$ as normalized factors, we define two random sequences as $\hat{W}_{n}=Z_{n}S_{n}^{-1}$, $\bar{W}_{n}=Z_{n}I_{n}^{-1}$, $n\in N$.

In what follows, we discuss the limit properties of $\{\hat{W}_{n},n\in N\}$ and $\{\bar{W}_{n},n\in N\}$.

## The limit properties of $\{\hat{W}_{n},n\in N\}$

### Theorem 5.1

$\{\hat{W}_{n},\mathfrak{F}_{n}(\vec{\xi}),n \in N \}$ *is a nonnegative supermartingale*, *and there exists a nonnegative finite random variable* *Ŵ* *such that*

$$ \lim_{n\rightarrow \infty}\hat{W}_{n}=\hat{W} \quad \textit{a.s.} $$

*and*

$$ E(\hat{W}|\vec{\xi})\leq 1. $$

### Proof

From Lemma 3.3 we obtain

5.1 $$ E\bigl(\hat{W}_{n+1}|\mathfrak{F}_{n}(\vec{\xi}) \bigr)=S_{n+1}^{-1}E(Z_{n+1}| \vec{ \xi})=S_{n+1}^{-1}Z_{n}m(\xi _{n})\alpha (\xi _{n})\varepsilon ( \xi _{n},Z_{n })\leq S_{n}^{-1}Z_{n}=\hat{W}_{n}, $$

namely $\{\hat{W}_{n},\mathfrak{F}_{n}(\vec{\xi}),n\in N \}$ is a nonnegative supermartingle. According to the Doob martingale convergence theorem, there exists a nonnegative, finite random variable *Ŵ* satisfying

$$ \lim_{n\rightarrow \infty}\hat{W}_{n}=\hat{W} \quad \text{a.s.} $$

Taking the conditional expectation with respect to *ξ* on both sides of of (5.1), we are able to obtain recursively

$$ E(\hat{W}_{n+1}|\vec{\xi})=E\bigl(E\bigl(\hat{W}_{n+1}| \mathfrak{F}_{n}(\vec{\xi})\bigr)|\vec{\xi}\bigr)\leq E( \hat{W}_{n}|\vec{\xi}) \leq \cdots \leq E(\hat{W}_{0}|\vec{ \xi})=1. $$

Using the Fatou Lemma gives

$$ E(\hat{W}|\vec{\xi})=E\Bigl(\liminf_{n\rightarrow \infty} \hat{W}_{n}\big|\vec{\xi}\Bigr)\leq \liminf_{n\rightarrow \infty}E( \hat{W}_{n}| \vec{\xi})\leq 1, $$

which completes the proof. □

### Theorem 5.2

*If* $\sum_{i=0}^{\infty}E( \frac{m_{2}(\xi _{i})}{S_{i}m^{2}(\xi _{i})\alpha (\xi _{i})\varepsilon (\xi _{i})})< \infty $ *and* $\sum_{i=0}^{\infty}E( \frac{\delta ^{2}(\xi _{i})}{S_{i}^{2}\varepsilon ^{2}(\xi _{i})})< \infty $, *then* $\{\hat{W}_{n},n\in N\}$ *is bounded in* $L^{2}$ *and converges in* $L^{1}$ *to* *Ŵ*.

### Proof

From Lemma 3.3 and the fact that for given *ξ⃗* and any $n\in N$, $k\in N^{+}$, $X_{nk}$ and $I_{nk}$ are mutually independent, one can derive

5.2 $$\begin{aligned} E\bigl(\hat{W}_{n+1}^{2}|\mathfrak{F}_{n}(\vec{ \xi})\bigr) =&S_{n+1}^{-2}E\bigl(Z_{n+1}^{2}| \mathfrak{F}_{n}(\vec{\xi})\bigr) \\ =&S_{n+1}^{-2}\bigl\{ Z_{n}\varepsilon (\xi _{n},Z_{n})\operatorname{Var}(X_{n1}I_{n1}| \vec{\xi})+m^{2}(\xi _{n})\alpha (\xi _{n})\bigl[ \delta ^{2}(\xi _{n},Z_{n}) \\ &{}+Z_{n}^{2}\varepsilon ^{2}(\xi _{n},Z_{n})\bigr]\bigr\} \\ \leq &\hat{W}_{n}^{2}+\hat{W}_{n} \frac{\operatorname{Var}(X_{n1}I_{n1}|\vec{\xi})}{S_{n}m^{2}(\xi _{n})\alpha ^{2}(\xi _{n})\varepsilon (\xi _{n})}+ \frac{\delta ^{2}(\xi _{n})}{S_{n}^{2}\varepsilon ^{2}(\xi _{n})} \\ \leq &\hat{W}_{n}^{2}+\hat{W}_{n} \frac{m_{2}(\xi _{n})}{S_{n}m^{2}(\xi _{n})\alpha (\xi _{n})\varepsilon (\xi _{n})}+ \frac{\delta ^{2}(\xi _{n})}{S_{n}^{2}\varepsilon ^{2 }(\xi _{n})}. \end{aligned}$$

Taking the conditional expectation on both sides of (5.2) and combining with Theorem 5.1, we have

5.3 $$\begin{aligned} E\bigl(\hat{W}_{n+1}^{2}|\vec{\xi}\bigr) =& E\bigl(E\bigl( \hat{W}_{n+1}^{2}|\mathfrak{F}_{n}(\vec{\xi})\bigr)| \vec{\xi}\bigr) \\ \leq &E\bigl(\hat{W}_{n}^{2}|\vec{\xi}\bigr)+ E\biggl( \biggl(\hat{W}_{n}\cdot \frac{m_{2}(\xi _{n})}{S_{n}\alpha (\xi _{n})\varepsilon (\xi _{n})m^{2}(\xi _{n})} +\frac{\delta ^{2}(\xi _{n})}{S^{2}_{n}\varepsilon ^{2}(\xi _{n})}\biggr)\Big| \vec{\xi}\biggr) \\ \leq &E\bigl(\hat{W}_{n}^{2}|\vec{\xi}\bigr)+ \frac{m_{2}(\xi _{n})}{S_{n}\alpha (\xi _{n})\varepsilon (\xi _{n})m^{2}(\xi _{n})}+ \frac{\delta ^{2}(\xi _{n})}{S^{2}_{n}\varepsilon ^{2}(\xi _{n})}. \end{aligned}$$

Taking the expectation on both sides of (5.3), it is deduced recursively that

5.4 $$ E\bigl(\hat{W}_{n+1}^{2}\bigr)\leq 1+E\Biggl(\sum _{i=0}^{n} \frac{m_{2}(\xi _{i})}{S_{i}\alpha (\xi _{i})\varepsilon (\xi _{i})m^{2}(\xi _{i})}\Biggr) +E\Biggl(\sum _{i=0}^{n} \frac{\delta ^{2}(\xi _{i })}{S^{2}_{i}\varepsilon ^{2}(\xi _{i})} \Biggr). $$

Owing to the assumed condition, we obtain that $\{E\hat{W}_{n}^{2},n\in N\}$ is bounded, namely $\{ \hat{W}_{n},n\in N\}$ is bounded in $L^{2}$. Hence, $\{ \hat{W}_{n},n\in N\}$ is uniformly integrable, which combined with Theorem 5.1 yields the desired result that $\{\hat{W}_{n},n\in N\}$ converges in $L^{1}$ to *Ŵ*. □

Now, we give the condition that the limit *Ŵ* of $\{\hat{W}_{n},n\in N\}$ is nondegenerate, beginning by introducing a Lemma.

### Lemma 5.3

([10])

*Set* $R^{+}=(0,+\infty )$, *when* *ξ⃗* *is given*, *for any fixed* $n\in N$,

(i) *If* $\{a_{j}(\xi _{n}),j\in N^{+}\}$ *is a nondecreasing sequence*, *there exists a nondecreasing function* $\varphi _{\xi _{n}}(\cdot )$ *on* $R^{+}$ *such that* $\varphi _{\xi _{n}}(x)\geq a_{1}(\xi _{n})$, $x>0$; $\varphi _{\xi _{n}}(j) \leq a_{j}(\xi _{n})$, $j\in N^{+}$ *and* $\varphi _{\xi _{n}}^{\ast}(x)\equiv x\cdot \varphi _{\xi _{n}}(x)$, $x>0$ *is convex*.
(ii) *If* $\{a_{j}(\xi _{n}),j\in N^{+}\}$ *is a nonincreasing sequence*, *there exists a nonincreasing function* $\psi _{\xi _{n}}(\cdot )$ *on* $R^{+}$ *such that* $\psi _{\xi _{n}}(x)\leq a_{1}(\xi _{n})$, $x>0$; $\psi _{\xi _{n}}(j)\geq a_{j}( \xi _{n})$, $j\in N^{+}$ *and* $\psi _{\xi _{n}}^{\ast}(x)\equiv x\cdot \psi _{\xi _{n}}(x)$, $x>0$ *is concave*.

For any fixed $n\in N$, let $\{\varepsilon (\xi _{n},k):k\in N^{+}\}$ be a nondecreasing sequence, then by Lemma 5.3 there exists a nondecreasing $\varphi _{\xi _{n}}(\cdot )$ on $R^{+}$ such that $\varphi _{\xi _{n}}(x)\geq \varepsilon (\xi _{n};1)$, $x>0$; $\varphi _{ \xi _{n}}(j)\leq \varepsilon (\xi _{n};j)$, $j\in N^{+}$ and $\varphi _{\xi _{n}}^{\ast}(x)\equiv x\varphi _{\xi _{n}}(x)$, $x>0$ is convex.

### Theorem 5.4

*For any fixed* $n\in N$, *if* $\{\varepsilon (\xi _{n};k):k\in N^{+}\} $ *is a nondecreasing sequence and*

$$ E\Biggl(\prod_{i=0}^{\infty} \frac{\varphi _{\xi _{i}} (N_{{0 }}\prod_{j=0}^{i-1}m(\xi _{j})\alpha (\xi _{j})\varepsilon _{1}(\xi _{j}))}{\varepsilon (\xi _{i})} \Biggr) >0, $$

*then* $E(\hat{W})>0$, *i*.*e*., $P(\hat{W}>0)>0$.

### Proof

From the Lemmas 3.3 and 5.3, one obtains

5.5 $$\begin{aligned} E\bigl(\hat{W}_{n+1}|\mathfrak{F}_{n}(\vec{\xi})\bigr) =&S_{n+1}^{-1}Z_{n}m( \xi _{n})\alpha ( \xi _{n})\varepsilon (\xi _{n};Z_{n}) \\ \geq &S_{n+1}^{-1}Z_{n}m(\xi _{n}) \alpha (\xi _{n})\varphi _{\xi _{n}}(Z_{n}) \\ =& S_{n+1}^{-1}m(\xi _{n})\alpha (\xi _{n})\varphi _{\xi _{n}}^{ \ast }(Z_{n})\quad \text{a.s.} \end{aligned}$$

Since for any $n\in N$, $\varphi _{\xi _{n}}(\cdot )$ is nondecreasing and $\varphi _{\xi _{n}}^{\ast}(\cdot )$ is convex, taking the conditional expectation on both sides of (5.5) and combining the Jensen inequality and Lemma 3.3 yields

5.6 $$\begin{aligned} E(\hat{W}_{n+1}|\vec{\xi}) \geq &S_{n+1}^{-1}m(\xi _{n})\alpha (\xi _{n}) E\bigl(\varphi _{\xi _{n}}^{\ast }(Z_{n})| \vec{\xi}\bigr) \\ \geq & S_{n+1}^{-1}m(\xi _{n})\alpha (\xi _{n})\varphi _{\xi _{n}}^{ \ast }\bigl(E(Z_{n}| \vec{\xi})\bigr) \\ =&S_{n+1}^{-1}m(\xi _{n})\alpha (\xi _{n})E(Z_{n}|\vec{\xi}) \varphi _{\xi _{n}} \bigl(E(Z_{n}|\vec{\xi})\bigr) \\ =&E(\hat{W}_{n}|\vec{\xi}) \frac{\varphi _{\xi _{n}}(E(Z_{n}|\vec{\xi}))}{\varepsilon (\xi _{n})} \\ \geq &E(\hat{W}_{n}|\vec{\xi}) \frac{\varphi _{\xi _{n}}(N_{{0 }}\prod_{i=0}^{n-1}m(\xi _{i})\alpha (\xi _{i})\varepsilon _{1}(\xi _{i}) )}{\varepsilon (\xi _{n})}. \end{aligned}$$

Iterating (5.6) with respect to *n*, we obtain

$$ E(\hat{W}_{n+1}|\vec{\xi})\geq \prod_{i=0}^{n} \frac{\varphi _{\xi _{i}}(N_{{0 }}\prod_{j=0}^{i-1}m(\xi _{j})\alpha (\xi _{j})\varepsilon _{1}(\xi _{j}))}{\varepsilon (\xi _{i})}. $$

By the assumed condition of Theorem 5.4 and Fatou Lemma, we deduce that

$$ \begin{aligned} E(\hat{W})&= E\Bigl(E\Bigl(\lim_{n\rightarrow \infty}\hat{W}_{n}\big| \vec{\xi}\Bigr)\Bigr) \geq E\Bigl(\limsup_{n\rightarrow \infty}E( \hat{W}_{n}|\vec{\xi})\Bigr)\\ &\geq E\Biggl( \prod _{i=0}^{\infty } \frac{\varphi _{\xi _{i}}(N_{{0 }}\prod_{j=0}^{i-1}m(\xi _{j})\alpha (\xi _{j})\varepsilon _{1}(\xi _{j}))}{\varepsilon (\xi _{i})}\Biggr), \end{aligned} $$

from which it follows $E(\hat{W})>0$, which completes the proof. □

### Theorem 5.5

*If* $P(\hat{W}>0)>0$, *then it holds on* $\{\hat{W}>0\}$

$$ \sum_{k=0}^{\infty}\biggl[1- \frac{\varepsilon (\xi _{k};Z_{n})}{\varepsilon (\xi _{k})} \biggr]< \infty ,\quad\textit{a.s.} $$

### Proof

For any $n\in N$, Lemma 3.3 implies

$$ E\bigl(\hat{W}_{n+1}|\mathfrak{F}_{n}(\vec{\xi}) \bigr)=S_{n+1}^{-1}Z_{n}m(\xi _{n}) \alpha (\xi _{n})\varepsilon (\xi _{n}; Z_{n})= \frac{\hat{W}_{n}\varepsilon (\xi _{n}; Z_{n})}{\varepsilon (\xi _{n} )}= \hat{W}_{n}- \frac{\hat{W}_{n}\varepsilon _{Z_{n }}(\xi _{n} )}{\varepsilon (\xi _{n} )}. $$

Hence,

5.7 $$ E(\hat{W}_{n+1})=E(\hat{W}_{n})- E\biggl( \frac{\hat{W}_{n}\varepsilon _{Z_{n }}(\xi _{n} )}{\varepsilon (\xi _{n} )} \biggr). $$

Since $E(\hat{W}_{0})=1$, iterating (5.7) gives

5.8 $$ E(\hat{W}_{n+1})=1-\sum_{k=0}^{n} E\biggl( \frac{\hat{W}_{k}\varepsilon _{Z_{k}}(\xi _{k} )}{\varepsilon (\xi _{k} )}\biggr) \geq 0 . $$

In (5.8), letting $n\rightarrow \infty $, we arrive at

$$ 0\leq \sum_{k=0}^{\infty} E\biggl( \frac{\hat{W}_{k}\varepsilon _{Z_{k}}(\xi _{k} )}{\varepsilon (\xi _{k} )}\biggr) \leq 1. $$

Thus,

5.9 $$ \sum_{k=0}^{\infty} \frac{\hat{W}_{k}\varepsilon _{Z_{k}}(\xi _{k} )}{\varepsilon (\xi _{k} )}< \infty \quad \text{a.s.} $$

From (5.9), we have for almost everywhere $w\in \{\hat{W}>0\}$, it holds that

$$ \sum_{k=0}^{\infty} \frac{\hat{W}_{k}(w)\varepsilon _{Z_{k}}(\xi _{k}(w) )}{\varepsilon (\xi _{k}(w) )}< \infty . $$

Since $\lim_{n\rightarrow \infty}\hat{W}_{n}(w)=\hat{W}(w)>0 $, by the sign-preserving property of the limit, there exists $k(w)$ satisfying $0< k(w)<\hat{W}(w)$ and $n_{0}(w)\in N^{+}$ such that when $n>n_{0}(w)$, it holds that

$$ k(w)\sum_{k=0}^{\infty}\biggl\{ 1- \frac{\varepsilon (\xi _{k}(w);Z_{k}(w) )}{\varepsilon (\xi _{k}(w))} \biggr\} \leq \sum_{k=0}^{\infty} \frac{\hat{W}_{k}(w)}{\varepsilon (\xi _{k}(w))}\bigl[\varepsilon \bigl(\xi _{k}(w)\bigr)- \varepsilon \bigl(\xi _{k}(w);Z_{k}(w)\bigr)\bigr]< \infty . $$

Therefore, on $\{\hat{W}>0\}$, we have $\sum_{k=0}^{\infty}[1- \frac{\varepsilon (\xi _{k};Z_{n})}{\varepsilon (\xi _{k})}]<\infty $, a.s. □

Below, we prove the convergence in $L^{2}$ of $\{\hat{W}_{n},n\in N\}$.

### Theorem 5.6

*Under the condition of Theorem *5.2, *if*

$$ \sum_{i=0}^{\infty}\biggl[E\biggl( \frac{\varepsilon (\xi _{i})-\varepsilon _{1}(\xi _{i})}{\varepsilon (\xi _{i})}\biggr)^{2 }\biggr]^{\frac{1}{2}}< \infty , \qquad \sum_{i=0}^{\infty} \Biggl[E\Biggl(\sum _{j=0}^{i-1} \frac{\delta ^{2}(\xi _{j})}{S_{j}^{2}\varepsilon ^{2 }(\xi _{j})} \Biggr) \Biggr]^{ \frac{1}{2}}< \infty $$

*and*

$$ \sum_{i=0}^{\infty} \Biggl[E\Biggl(\sum _{j=0}^{i-1} \frac{m_{2}(\xi _{j})}{S_{j}m^{2}(\xi _{j})\alpha (\xi _{j})\varepsilon (\xi _{j})} \Biggr) \Biggr]^{\frac{1}{2}}< \infty . $$

*Then*, $\{\hat{W}_{n},n\in N\} $ *converge in* $L^{2}$ *to* *Ŵ*.

### Proof

Since $\{\hat{W}_{n},\mathfrak{F}_{n}(\vec{\xi}),n\in N\}$ is a nonnegative supermartingale, from the Doob martingale decomposition theorem, it follows that, for any $n\in N$, $\hat{W}_{n}=Y_{n}-T_{n}$, where $\{Y_{n},\mathfrak{F}_{n}(\vec{\xi}),n\in N\}$ is a martingale, $\{T_{n},n\in N\}$ is an increasing process with

$$ T_{0}=0,\qquad T_{n}=\sum_{i=0}^{n-1} \frac{\hat{W}_{i}\varepsilon _{Z_{i }}(\xi _{i})}{\varepsilon (\xi _{i})}. $$

In what follows, we prove $\{T_{n},n\in N\}$ is bounded in $L^{2}$.

Since

$$ \Vert T_{n} \Vert _{2}= \Biggl\Vert \sum _{i=0}^{n-1} \frac{\hat{W}_{i}\varepsilon _{Z_{i }}(\xi _{i})}{\varepsilon (\xi _{i})} \Biggr\Vert _{2} \leq \sum_{i=0}^{n-1} \biggl\Vert \frac{\hat{W}_{i}\varepsilon _{Z_{i }}(\xi _{i})}{\varepsilon (\xi _{i})} \biggr\Vert _{2} = \sum _{i=0}^{n-1}\biggl[E\biggl( \frac{\hat{W}_{i}^{2}\varepsilon _{Z_{i }}^{2}(\xi _{i})}{\varepsilon ^{2}(\xi _{i})}\biggr) \biggr]^{ \frac{1}{2 }}, $$

from (5.4) we can derive

$$\begin{aligned} E\biggl( \frac{\hat{W}_{i}^{2}\varepsilon _{Z_{i }}^{2}(\xi _{i})}{\varepsilon ^{2}(\xi _{i})}\Big| \vec{\xi}\biggr) \leq & E\biggl( \hat{W}_{i}^{2}\biggl( \frac{\varepsilon (\xi _{i})-\varepsilon _{1}(\xi _{i})}{\varepsilon (\xi _{i})} \biggr)^{2}\Big| \vec{\xi}\biggr) \\ =&\biggl( \frac{\varepsilon (\xi _{i})-\varepsilon _{1}(\xi _{i})}{\varepsilon (\xi _{i})}\biggr)^{2}E\bigl( \hat{W}_{i}^{2}| \vec{\xi}\bigr) \\ \leq &\biggl( \frac{\varepsilon (\xi _{i})-\varepsilon _{1}(\xi _{i})}{\varepsilon (\xi _{i})}\biggr)^{2} \Biggl\{ 1+\sum _{j=0}^{i-1}\biggl[ \frac{m_{2}(\xi _{j})}{S_{j}\alpha (\xi _{j})\varepsilon (\xi _{j})m^{2}(\xi _{j})}+ \frac{\delta ^{2}(\xi _{j})}{S_{j}^{2}\varepsilon ^{2}(\xi _{j}) }\biggr] \Biggr\} . \end{aligned}$$

Thus,

$$ \begin{aligned} E\biggl( \frac{\hat{W}_{i}^{2}\varepsilon _{Z_{i }}^{2}(\xi _{i})}{\varepsilon ^{2}(\xi _{i})}\biggr) &\leq E\biggl(\biggl( \frac{\varepsilon (\xi _{i})-\varepsilon _{1}(\xi _{i})}{\varepsilon (\xi _{i})} \biggr)^{2} \biggr)+\sum_{j=0}^{i-1}E \biggl( \frac{m_{2}(\xi _{j})}{S_{j}\alpha (\xi _{j})\varepsilon (\xi _{j})m^{2}(\xi _{j}) }\biggr)\\ &\quad {}+ \sum_{j=0}^{i-1}E \biggl( \frac{\delta ^{2}(\xi _{j})}{S_{j}^{2}\varepsilon ^{2}(\xi _{j})}\biggr). \end{aligned} $$

Therefore,

$$\begin{aligned} \Vert T_{n} \Vert _{2 } \leq & \sum _{i=0}^{n-1}\biggl[E\biggl(\biggl( \frac{\varepsilon (\xi _{i})-\varepsilon _{1}(\xi _{i})}{\varepsilon (\xi _{i})} \biggr)^{2} \biggr) \biggr]^{\frac{1}{2 }} +\sum _{i=0}^{n-1}\Biggl[E\Biggl(\sum _{j=0}^{i-1} \frac{m_{2}(\xi _{j})}{S_{j}\alpha (\xi _{j})\varepsilon (\xi _{j})m^{2}(\xi _{j}) }\Biggr) \Biggr]^{ \frac{1}{2 }} \\ &{}+\sum_{i=0}^{n-1}\Biggl[E\Biggl(\sum _{j=0}^{i-1} \frac{\delta ^{2}(\xi _{j})}{S_{j}^{2}\varepsilon ^{2}(\xi _{j})}\Biggr) \Biggr]^{ \frac{1}{2}}. \end{aligned}$$

According to the assumed condition of Theorem 5.6, $\{T_{n},n\in N\}$ is bounded in $L^{2}$, from which and the fact $\{T_{n},n\in N\}$ is a nonnegative increasing process, it follows that $\{T_{n},n\in N\}$ converges in $L^{2}$. From Theorem 5.2, we have $\{\hat{W}_{n},n\in N\}$ is bounded in $L^{2}$, so $\{Y_{n},n\in N\}$ is bounded in $L^{2}$. Since $\{Y_{n},\mathfrak{F}_{n}(\vec{\xi} ),n\in N\} $ is a martingale, $\{Y_{n},n\in N\}$ converges in $L^{2}$, and therefore $\{\hat{W}_{n},n\in N\}$ converges in $L^{2}$ to *Ŵ*. □

## The limit properties of $\{\bar{W}_{n},n\in N\}$

### Theorem 6.1

*If* $E(\prod_{k=0}^{\infty} \frac{\varepsilon (\xi _{k})}{\varepsilon _{1}(\xi _{k})})<\infty$, *then there exists a nonnegative*, *infinite random variable* *W̄* *such that* $\lim_{n\rightarrow \infty} \bar{W}_{n}=\bar{W}$ *a*.*s*., *and* $E(\bar{W})<\infty $.

### Proof

From Lemma 3.3, it follows that

$$\begin{aligned} E\bigl(\bar{W}_{n+1}|\mathfrak{F}_{n}(\vec{\xi}) \bigr) =&I_{n+1}^{-1}E\bigl(Z_{n+1}| \mathfrak{F}_{n}(\vec{\xi})\bigr)=I_{n+1}^{-1}Z_{n}m( \xi _{n})\alpha (\xi _{n}) \varepsilon (Z_{n}; \xi _{n}) \\ =&\bar{W}_{n}\cdot \frac{\varepsilon (Z_{n}; \xi _{n})}{\varepsilon _{1}(\xi _{n})}\geq \bar{W}_{n}. \end{aligned}$$

Namely, $\{\bar{W}_{n},\mathfrak{F}_{n}(\vec{\xi}),n\in N\}$ is a nonnegative submartingale and

6.1 $$ E(\bar{W}_{n}|\vec{\xi})=E\bigl(I_{n}^{-1}Z_{n}| \vec{\xi}\bigr)\leq I_{n}^{-1}N_{0} \prod _{i=0}^{n-1}m(\xi _{i})\alpha (\xi _{i})\varepsilon ( \xi _{i}) \leq \prod _{i=0}^{n-1} \frac{\varepsilon ( \xi _{i})}{\varepsilon _{1}( \xi _{i})}. $$

Taking expectation on both sides of (6.1), we arrive at

$$ E(\bar{W}_{n})\leq E\Biggl(\prod_{i=0}^{n-1} \frac{\varepsilon ( \xi _{i})}{\varepsilon _{1}( \xi _{i})} \Biggr). $$

An immediate consequence of the assumed condition of Theorem 6.1 is $\sup_{n\geq 0}E(\bar{W}_{n})<\infty $. By the submartingale convergence theorem, there exists a nonnegative random variable *W̄* such that

$$ \lim_{n\rightarrow \infty}\bar{W}_{n}=\bar{W},\quad\text{a.s.} $$

and $E(\bar{W})<\infty $.

Below, we discuss the condition of $\{\bar{W}_{n},n\in N\} $ converges in $L^{1}$.

We set

$$ r_{k}(\xi _{n})=k^{-1}E\bigl( \bigl\vert Z_{n+1}-k\varepsilon _{1}(\xi _{n})m(\xi _{n}) \alpha (\xi _{n}) \bigr\vert |Z_{n}=k, \mathfrak{F}_{n}(\vec{\xi})\bigr),\quad k\in N^{+},n \in N, $$

then it holds that $E(\frac{|\bar{W}_{n+1}-\bar{W}_{n}|}{\bar{W}_{n}}|Z_{n},\mathfrak{F}_{n}( \vec{\xi}))=(\varepsilon _{1}(\xi _{n})m(\xi _{n}))^{-1}r_{Z_{n}}( \xi _{n})$. for fixed $n\in N$, let $\{r_{k}(\xi _{n}),k\in N^{+}\}$ be a nonincreasing sequence. Namely, as the number of particles increases, the absolute value of the average growth rate of $\bar{W}_{n}$ is required to decrease. By Lemma 5.3, there exists a nonincreasing function $\psi _{\xi _{n}}(\cdot )$ on $R^{+}$ such that $\psi _{\xi _{n}}(x)\leq r_{1}(\xi _{n})$, $x>0$; $\psi _{\xi _{n}}(j)\geq r_{j}( \xi _{n})$, $j\in N^{+}$ and $\psi _{\xi _{n}}^{\ast }(x)\equiv x\psi _{\xi _{n}}(x)$, $x>0$ is concave. □

### Lemma 6.2

*Suppose*

$$ \sum_{n=0}^{\infty}E\Biggl(\prod _{k=0}^{n-1} \frac{\varepsilon (\xi _{k})}{\varepsilon _{1}(\xi _{k})}\cdot \frac{\psi _{\xi _{n}}(N_{0}\prod_{i=0}^{n-1}m(\xi _{i})\alpha (\xi _{i})\varepsilon _{1}(\xi _{i}))}{m(\xi _{n})\alpha (\xi _{n })\varepsilon _{1}(\xi _{n })} \Biggr)< \infty $$

*and for given* *n*, $\{r_{k}(\xi _{n}):k\in N^{+}\}$ *is a nonincreasing sequence*, *then* $\{\bar{W}_{n},n\in N\}$ *converges in* $L^{1}$ *to nonnegative*, *infinite random variable* *W̄*.

### Proof

We begin with proving $\{\bar{W}_{n},n\in N\} $ is a $L^{1}\text{-}\mathit{Cauchy}$ sequence. By considering Lemma 5.3, it suffices to show that

$$\begin{aligned} E\bigl( \vert \bar{W}_{n+1}-\bar{W}_{n} \vert |\vec{\xi} \bigr) =&I_{n+1}^{-1}E\bigl( \bigl\vert Z_{n+1}- \varepsilon _{1}(\xi _{n })m(\xi _{n })\alpha (\xi _{n })Z_{n} \bigr\vert \vec{\xi}\bigr) \\ =&I_{n+1}^{-1}E\bigl(E\bigl[ \bigl\vert Z_{n+1}- \varepsilon _{1}(\xi _{n })m(\xi _{n }) \alpha (\xi _{n })Z_{n} \bigr\vert \mathfrak{F}_{n}(\vec{ \xi})\bigr]|\vec{\xi}\bigr) \\ =&I_{n+1}^{-1}E\bigl(Z_{n}r_{Z_{n}}(\xi _{n })|\vec{\xi}\bigr)\leq I_{n+1}^{-1}E \bigl(Z_{n} \psi _{\xi _{n}}(Z_{n })|\vec{\xi}\bigr) \\ =&I_{n+1}^{-1}E\bigl(\psi _{\xi _{n}}^{\ast }(Z_{n})| \vec{\xi}\bigr). \end{aligned}$$

Since $\psi _{\xi _{n}}(\cdot )$ is nondecreasing and $\psi _{\xi _{n}}^{\ast}(\cdot )$ is concave, then by Jensen’s inequality, we obtain

$$\begin{aligned} E\bigl( \vert \bar{W}_{n+1}-\bar{W}_{n} \vert |\vec{\xi} \bigr) \leq & I_{n+1}^{-1} \psi _{\xi _{n}}^{\ast} \bigl(E( Z_{n}|\vec{\xi})\bigr) =I_{n+1}^{-1}E( Z_{n}| \vec{\xi})\psi _{\xi _{n}} \bigl(E( Z_{n}|\vec{ \xi})\bigr) \\ =& \frac{E( \bar{W}_{n}|\vec{\xi})\psi _{\xi _{n}} (E( Z_{n}|\vec{\xi}))}{\varepsilon _{1}(\xi _{n })m(\xi _{n })\alpha (\xi _{n })}. \end{aligned}$$

Lemma 3.3 implies that

$$\begin{aligned} E\bigl( \vert \bar{W}_{n+1}-\bar{W}_{n} \vert |\vec{\xi} \bigr) \leq & \frac{\psi _{\xi _{n}}(N_{0}\prod_{i=0}^{n-1}\varepsilon (\xi _{i})m(\xi _{i})\alpha (\xi _{i}))}{\varepsilon _{1}(\xi _{n })m(\xi _{n })\alpha (\xi _{n })} \cdot \frac{\prod_{i=0}^{n-1}\varepsilon (\xi _{i})m(\xi _{i})\alpha (\xi _{i})}{ \prod_{i=0}^{n-1}\varepsilon _{1}(\xi _{i})m(\xi _{i})\alpha (\xi _{i})} \\ =& \frac{\psi _{\xi _{n}}( N_{0}\prod_{i=0}^{n-1}\varepsilon (\xi _{i})m(\xi _{i})\alpha (\xi _{i}))}{\varepsilon _{1}(\xi _{n })m(\xi _{n })\alpha (\xi _{n })} \cdot \prod_{i=0}^{n-1} \frac{\varepsilon (\xi _{i})}{\varepsilon _{1}(\xi _{i})}. \end{aligned}$$

Thus, we have

6.2 $$ E\bigl( \vert \bar{W}_{n+1}-\bar{W}_{n} \vert \bigr)\leq E\Biggl( \frac{\psi _{\xi _{n}}( N_{0}\prod_{i=0}^{n-1}\varepsilon (\xi _{i})m(\xi _{i})\alpha (\xi _{i}))}{\varepsilon _{1}(\xi _{n })m(\xi _{n })\alpha (\xi _{n })} \cdot \prod_{i=0}^{n-1} \frac{\varepsilon (\xi _{i})}{\varepsilon _{1}(\xi _{i})}\Biggr). $$

Summing (6.2) with respect to *n* gives

$$ \sum_{n=0}^{\infty}E\bigl( \vert \bar{W}_{n+1}-\bar{W}_{n} \vert \bigr)\leq \sum _{n=0}^{ \infty} E\Biggl( \frac{\psi _{\xi _{n}}( N_{0}\prod_{i=0}^{n-1}\varepsilon (\xi _{i})m(\xi _{i})\alpha (\xi _{i}))}{\varepsilon _{1}(\xi _{n })m(\xi _{n })\alpha (\xi _{n })} \cdot \prod _{i=0}^{n-1} \frac{\varepsilon (\xi _{i})}{\varepsilon _{1}(\xi _{i})}\Biggr). $$

Considering the assumed condition of Lemma 6.2, it is immediately clear that

$$ \lim_{n\rightarrow \infty}E\bigl( \vert \bar{W}_{n+1}- \bar{W}_{n} \vert \bigr)=0. $$

Namely, $\{\bar{W}_{n},n\in N\}$ is a $L^{1}\text{-}\mathit{Cauchy}$ sequence, so $\{\bar{W}_{n},n\in N\} $ converges in $L^{1}$ to a nonnegative, finite random variable *W̄*. □

## Conclusion

A model of branching processes with random control functions and affected by viral infectivity in an i.i.d. random environment is established, and the Markov property of the model, the sufficient conditions for certain extinction, and some limit properties of the normalized processes are studied. The relevant conclusions of the branching processes are extended and their application fields are expanded. Next, we intend to study the limit theory of the model further, such as the convergence rate of the limit and the central limit theorem, and some properties of the branching processes with random control functions and affected by viral infectivity in i.i.d. random environments with different distributions and stationary traversal random environments, and will try to give application examples.

## Data Availability

Data sharing is not applicable to this article as no datasets were generated or analyzed during the current study.
